# Supplementary material for: Crystal structure of steroid reductase SRD5A reveals conserved steroid reduction mechanism
Source: Nat Commun. 2021 Jan 19;12:449. doi: 10.1038/s41467-020-20675-2 (PMC7815742; doi:10.1038/s41467-020-20675-2)
Supplement: Supplementary file 1 — Supplementary Information [file 41467_2020_20675_MOESM1_ESM.pdf]

Supplementary Information for

Crystal structure of steroid reductase SRD5A reveals conserved steroid reduction mechanism

Yufei Han[1†], Qian Zhuang[2†], Bo Sun[3†], Wenping Lv[4†], Sheng Wang[5†], Qingjie Xiao[6], Bin Pang[1], Youli Zhou[1], Fuxing Wang[1], Pengliang Chi[6], Qisheng Wang[3], Zhen Li[7], Lizhe Zhu[4], Fuping Li[8], Dong Deng[6*], Ying-Chih Chiang[1*], Zhenfei Li[2*], Ruobing Ren[1*]

[1] Kobilka Institute of Innovative Drug Discovery, School of Life and Health Sciences, The Chinese University of Hong Kong, Shenzhen, Guangdong 518172, China

[2] State Key Laboratory of Cell Biology, Shanghai Institute of Biochemistry and Cell Biology, Center for Excellence in Molecular Cell Science, Chinese Academy of Sciences; University of Chinese Academy of Sciences, 320 Yueyang Road, Shanghai 200031, China

[3] Shanghai Synchrotron Radiation Facility, Shanghai Advanced Research Institute, Chinese Academy of Sciences, Shanghai 201204, China

[4] Warshel Institute for Computational Biology, School of Life and Health Sciences, The Chinese University of Hong Kong, Shenzhen, Guangdong 518172, China

[5] Tencent AI lab, Shenzhen, Guangdong 518000, China

[6] Department of Obstetrics, Key Laboratory of Birth Defects and Related Disease of Women and Children of MOE, State Key Laboratory of Biotherapy, West China Second Hospital, Sichuan University, Chengdu 610041, China

[7] School of Science and Engineering, The Chinese University of Hong Kong, Shenzhen, Guangdong 518172, China

[8] Human Sperm Bank, Key Laboratory of Birth Defects and Related Disease of Women and Children of MOE, West China Second Hospital, Sichuan University, Chengdu 610041, China

* To whom correspondence should be addressed. Email: renruobing@cuhk.edu.cn, zhenfei.li@sibcb.ac.cn, chiangyc@cuhk.edu.cn, and dengd@scu.edu.cn

† These authors contribute equally

This PDF File contains:

Supplementary Figures 1-10

Supplementary Tables 1-4

Supplementary Figures 1-9

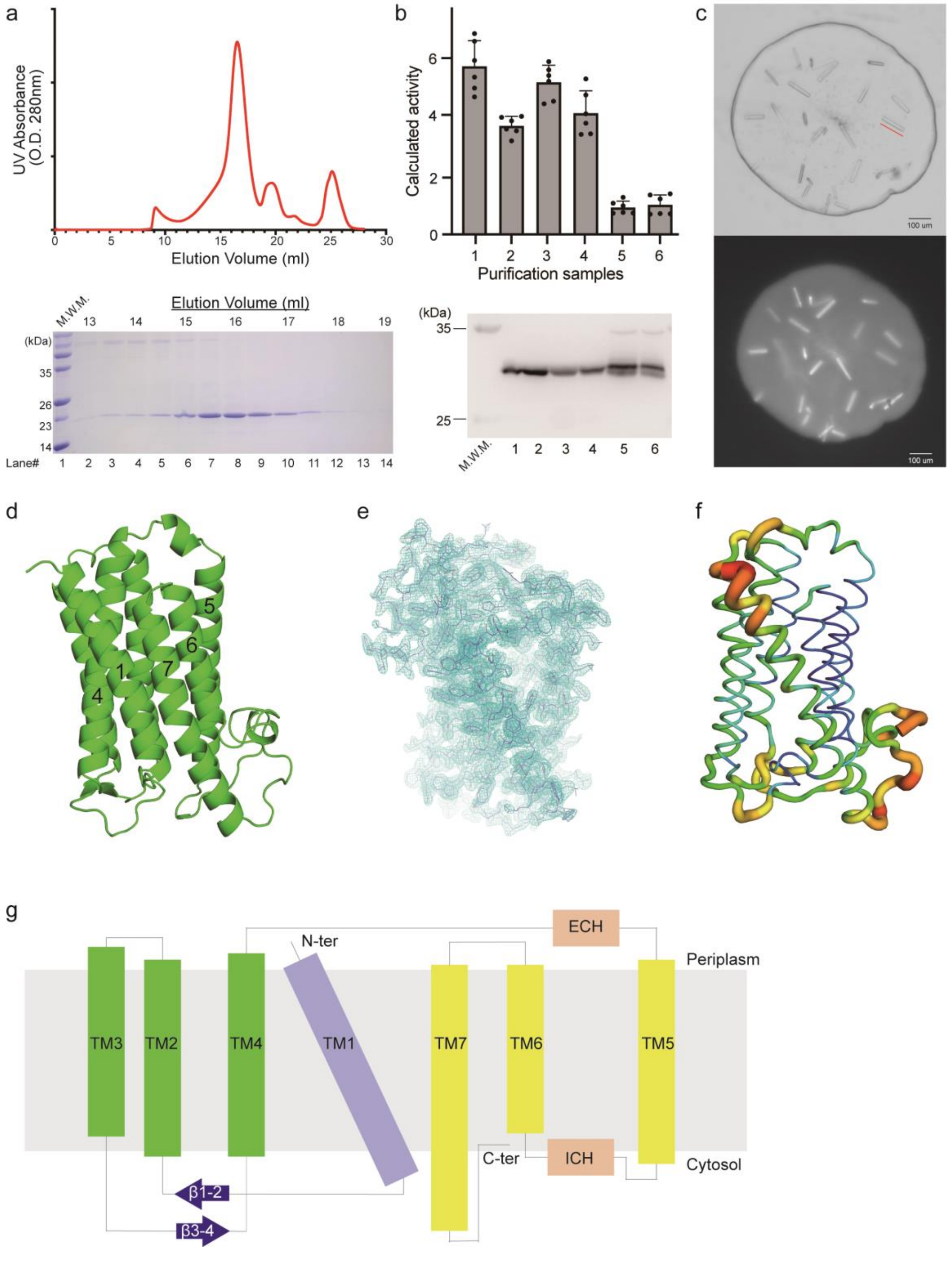

Supplementary Fig. 1 | Purification, crystallization and structural determination of PbSRD5A. a, The representative size exclusion chromatography of PbSRD5A and SDS-PAGE gel stained by Coomassie Brilliant Blue R-250. **b,** The enzyme activity of PbSRD5A during purification. The samples include: (1) whole cell crude extract; (2)

re-suspended cell membrane after 1st round of ultracentrifugation; (3) solubilized solution in 1% DDM; (4) supernatant after 2nd round of ultracentrifugation, (5) protein eluted from Ni-column, and (6) purified protein after size-exclusion chromatography. HPLC was used to detect the 5a-DHP% converted from progesterone, using the same units of protein. Equally protein was quantified by calculating the grey level of western blot. The activities were calculated as: *Calculated activity* = *detected activity* / (*Sample volumes in an assay system* × *grey value* / *Volume loaded on western blot*), and normalized by sample (6). Data are mean±s.d. derived from technically independent experiments in duplicate. Each experiment was reproduced three times on separate occasions with similar results. **c,** Photograph of PbSRD5A crystals in lipidic cubic phase under visible (upper) and UV light (lower). **d,** The initial searching model of PbSRD5A for molecular replacement is shown as green cartoon. **e,** The $2F_o$-F_c electron density map of PbSRD5A (cyan mesh) is contoured at 1.2σ. **f,** The b factor of PbSRD5A is colored by rainbow. Red and blue represent the highest and lowest b factor values, respectively. **g,** Topology of PbSRD5A. The seven transmembrane segments are divided into TM1 (lightblue), TM2-4 (green), and TM5-7 (yellow). The extracellular and intracellular short alpha helices (ECH and ICH) are colored wheat. The short anti-parallel beta strands are colored blue.

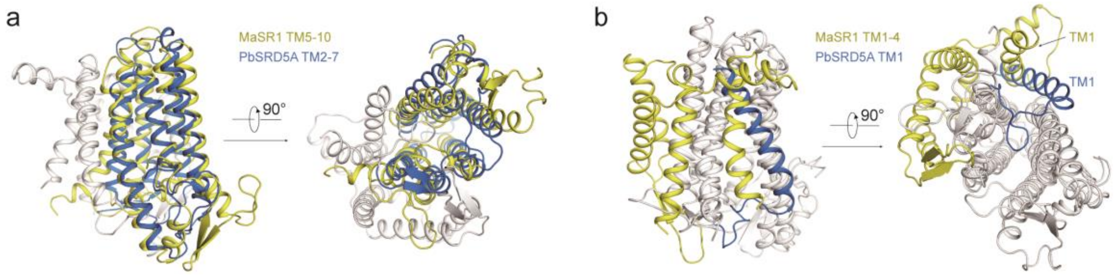

Supplementary Fig. 2 | Structural alignment of PbSRD5A and MaSR1. a, The superposition of TM2-7 of PbSRD5A and TM5-10 of MaSR1 is generated using cealign in pymol. TM2-7 of PbSRD5A and TM5-10 of MaSR1 (PDB code: 4QUV) are colored marine and yellow, respectively. TM1 of PbSRD5A and TM1-4 of MaSR1 are colored white. Two perpendicular views are shown. TM2-7 of PbSRD5A and TM5-10 of MaSR1 are superimposed with the r.m.s.d. of 3.60 Å over Cα of 184 residues. **b,** TM1 of PbSRD5A and TM1-4 of MaSR1 are colored marine and yellow, respectively. TM2-7 of PbSRD5A and TM5-10 of MaSR1 are colored white. Two TM1s adopt distinct conformations and TM2-4 of MaSR1 is missing in PbSRD5A.

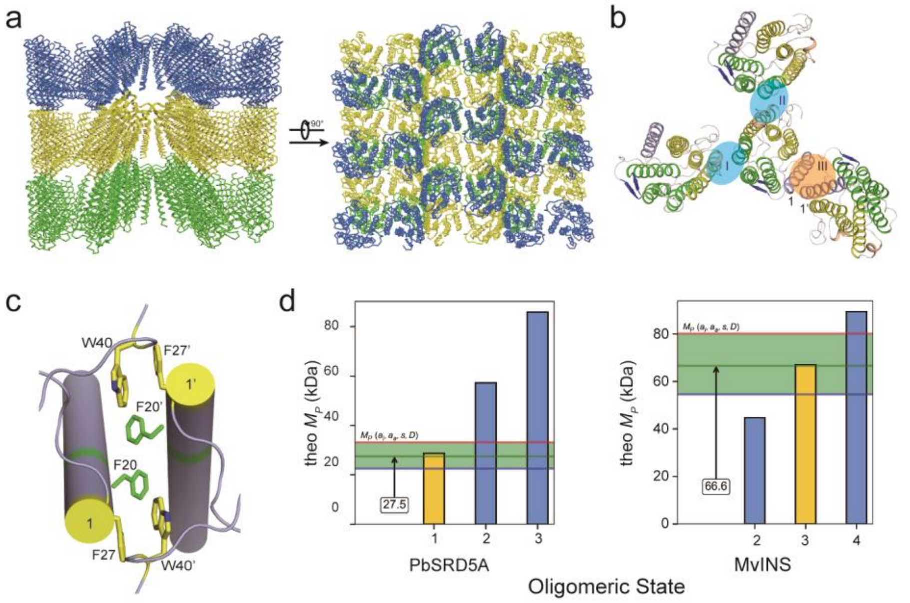

Supplementary Fig. 3 | Crystal packing and oligomerization state examination of PbSRD5A. a, Two perpendicular views of the crystal packing of PbSRD5A in the space group of $C222_1$. **b,** One PbSRD5A molecule interacts with three adjacent molecules in the crystal. The interface I and II are highlighted in cyan shadow and interface III is in orange. **c,** The hydrophobic residues involved in interface III are shown as yellow and green sticks. **d,** The estimated molecular weights were calculated by AUC analysis. Ranges of PbSRD5A and MvINS are shown in palegreen. X-axis values are the putative oligomerization states.

a

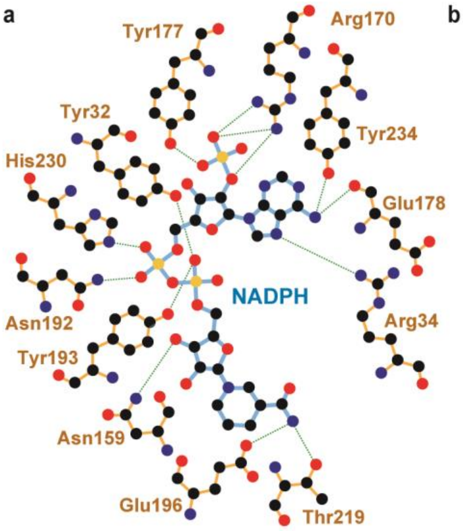

b **PbSRD5A-NADPH Hydrogen Bond Networks by PISA**

No.	NADPH	Distance (Å)	PbSRD5A
1	N6A	2.68	Glu178 [O]
2	N6A	3.05	Tyr234 [OH]
3	N7A	2.83	Arg34 [NH2]
4	N7N	2.98	Glu196 [OE1]
5	N7N	3.21	Thr219 [OG1]
6	O1A	2.93	Asn192 [ND2]
7	O1N	2.78	Tyr32 [OH]
8	O1N	2.48	Tyr193[OH]
9	O1N	3.51	Arg226 [NE]
10	O1X	2.60	Tyr177 [OH]
11	O2A	3.19	Tyr193 [OH]
12	O2A	2.80	His230 [NE2]
13	O2B	2.77	Arg170 [NH1]
14	O2D	3.41	Asn159 [ND2]
15	O2X	2.93	Arg170 [NH2]
16	O2X	2.76	Arg170 [NH1]
17	O3	3.49	Asn192 [ND2]
18	O3B	3.21	Arg99 [O]
19	O3D	2.98	Arg90 [NH2]
20	O3D	2.78	Asn159 [ND2]
21	O3D	2.63	Asp163 [OD2]
22	O7N	3.51	Asn222 [ND2]

c **Other PbSRD5A-NADPH Interactions by LigPlot+**

No.	PbSRD5A	Interactions	No.	PbSRD5A	NADPH	Interactions
1	Trp50	Hydrophobic interaction	1	Arg34 [NE]	O1X	Water mediated hydrogen bonds
2	Phe94	Hydrophobic interaction	2	Val100 [O]	O3X	Water mediated hydrogen bonds
3	Met98	Hydrophobic interaction	3	Leu166 [O]	N3A	Water mediated hydrogen bonds
4	Leu166	Hydrophobic interaction	4	Asn192 [O]	O1A	Water mediated hydrogen bonds
5	Leu169	Hydrophobic interaction	5	Arg226 [NH2]	O2A	Water mediated hydrogen bonds
6	Leu223	Hydrophobic interaction				

Supplementary Fig. 4 | a, Residues that directly form hydrogen bonds with NADPH are shown in stick-ball model. The bonds of residues and NADPH are colored in yellow and cyan, respectively. The atoms are colored by elements (black for carbon, red for oxygen, purple for nitrogen, and yellow for phosphorus). The diagram is generated by LigPlot+[1]. **b,** The hydrogen bond distances between atoms in PbSRD5A residues and NADPH are measured by PISA[2]. The residues shown in panel **a** are colored in brown. Other hydrogen bonds only found by PISA are colored in black. **c,** The indirect interactions between PbSRD5A and NADPH were listed as two categories. The hydrophobic interactions and water mediated polar interactions are listed in the left and right panels, respectively. These interactions are analyzed by LigPlot+[1].

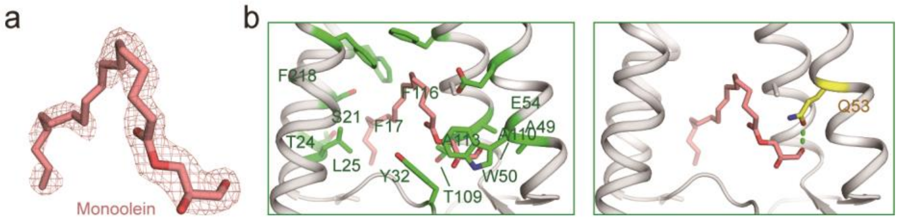

Supplementary Fig. 5 | PbSRD5A accommodates one MAG molecule in crystal structure. a, Monoolein fits into the density map shown in Fig. **2b**. **b,** Coordination of monoolein in PbSRD5A. The residues that interact with monoolein with hydrophobic effect, such as F17, S21 T24, L25, Y32, A49, W50, T109, A110, A113 and F116, are shown as green sticks. Q53, shown as yellow sticks, forms hydrogen bond (green dash) with the hydroxyl group of monoolein.

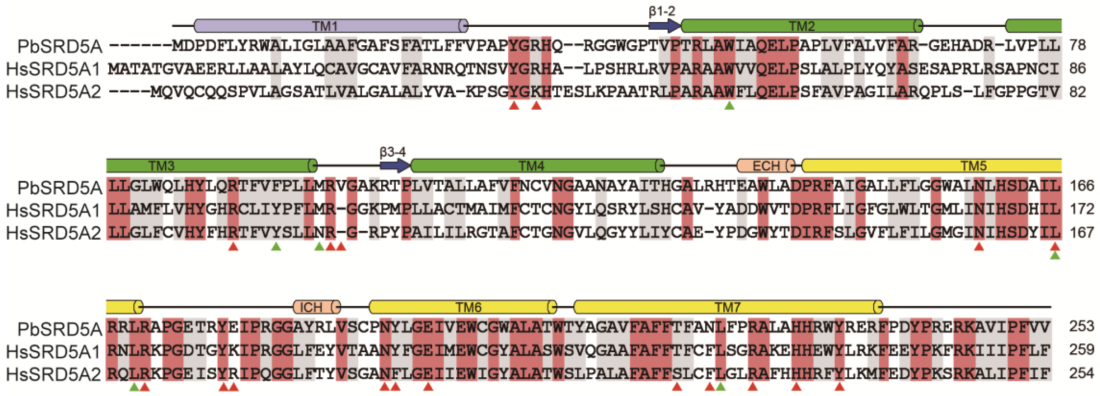

Supplementary Fig. 6 | Sequence alignment of PbSRD5A with HsSRD5A1 and -2.

Secondary structural elements of PbSRD5A are indicated above the sequence alignment. Invariant and highly conserved amino acids are shaded in rose red and grey, respectively. The residues identified for NADPH binding are highlighted at the bottom by red (polar interactions) and green triangles (hydrophobic effects), respectively.

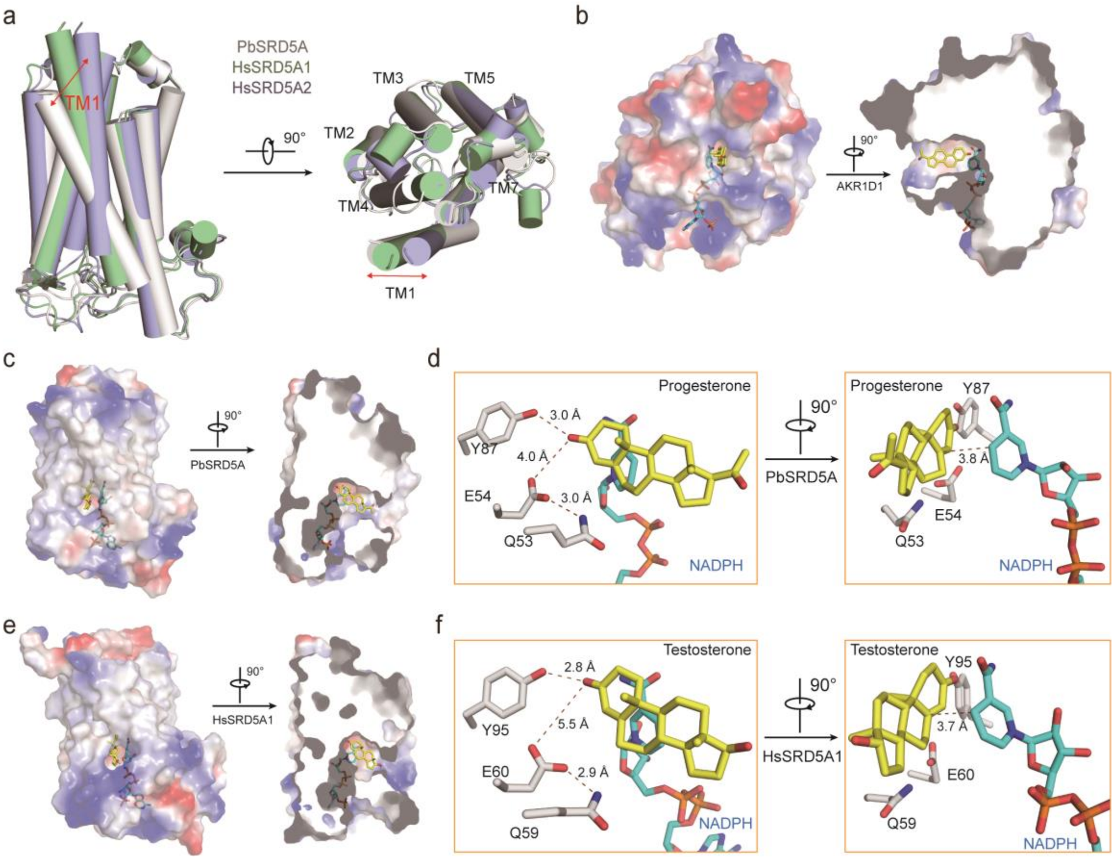

Supplementary Fig. 7 | Structural comparison and substrate docking of SRD5As. **a,** The superposition of PbSRD5A (gray), HsSRD5A1 (palegreen) and HsSRD5A2 (lightblue) is shown as cylindrical cartoon. The arrows indicate the conformational difference of TM1s in SRD5As by two perpendicular views. **b,** The structure of AKR1D1-progesterone (PDB code: 3COT). The semi-transparent electrostatic surface of HsSRD5A2 is shown. **c,** The docking pose of progesterone in PbSRD5A docking model. **d,** The coordination of conserved Q-E-Y motif with progesterone in PbSRD5A docking model. **e,** The docking pose of progesterone in HsSRD5A1 docking model. **f,** The coordination of conserved Q-E-Y motif in HsSRD5A1 docking model. In (**b**)-(**f**), substrates are shown as yellow stick. NADPH is colored cyan. Two perpendicular views are shown.

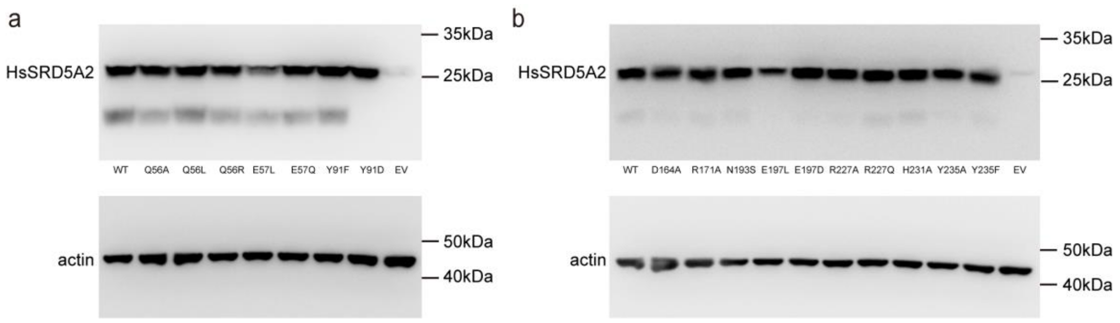

Supplementary Fig. 8 | The expression level of HsSRD5A2 mutants in HEK293 cells. a, The western blot for WT, Q56A, Q56L, Q56R, E57L, E57Q, Y91F, Y91D and EV in Fig. 3f. **b,** The western blot for WT, D164A, R171A, N193S, E197L, E197D, R227A, R227Q, H231A, Y235A, Y235F and EV in Fig. 4b.

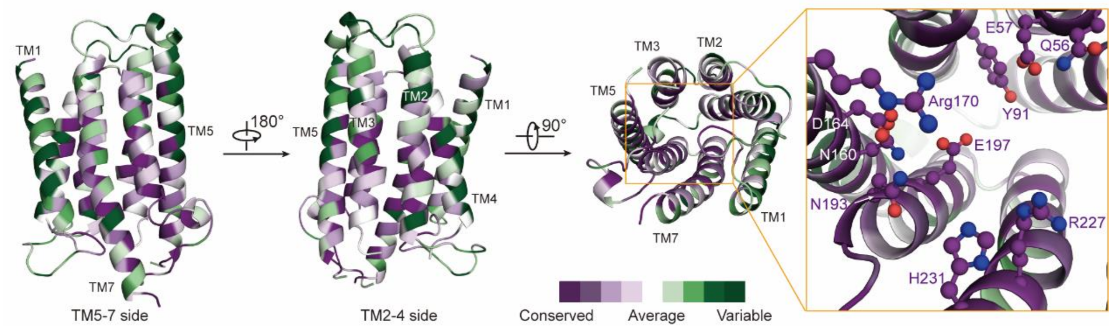

Supplementary Fig. 9 | Mapping of the conserved residues of steroid 5-alpha reductases to the structural model of HsSRD5A2. Sequence alignment was made for 150 steroid 5-alpha reductases and putative homologues. The conserved residues were mapped to the structural model of HsSRD5A2 using ConSurf[3,4]. Invariant residues in NADPH binding and catalytic site residues are show in stick-ball model in the inset.

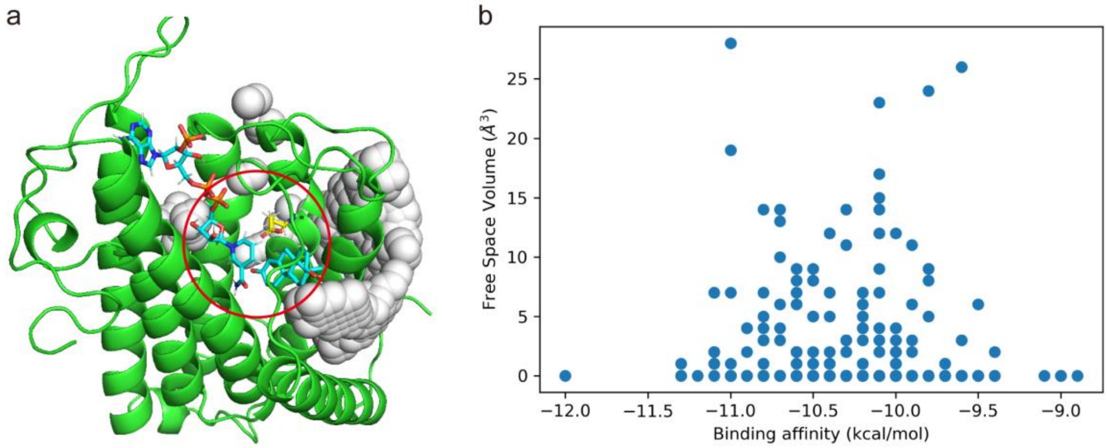

Supplementary Fig. 10 | The extra space in substrate binding pocket of HsSRD5A2 is limited to accommodate water molecule in the presence of substrate. a, An oxygen atom is used as a probe to search the volume of free space around E57 (radius of 15Å to the carbon atom of the carboxyl group of E57) at the optimal binding pose. The free space is shown in white spheres. Testosterone (cyan), NADPH (cyan), and E57 (yellow) are shown in stick mode. HsSRD5A2 is shown in cartoon mode. **b,** A correlation analysis of the free space volume around E57 after testosterone is docked and the predicted binding affinity of testosterone.

Supplementary Tables 1-4

Samples	(1)	(2)	(3)	(4)	(5)	(6)
Purification volume (mL)	50	40	55	50	16	2.5
Assay volumes (μL)	0.30	0.24	0.33	0.30	0.96	0.15
Total protein （mg）	-	-	-	857.50	1.60	0.15
Total activity (μmol·min^{-1})	6.85×10^{-6}	5.34×10^{-6}	5.93×10^{-6}	4.82×10^{-6}	8.75×10^{-7}	5.27×10^{-7}
**Specific activity (μmol·mg^{-1}·min^{-1})**	**-**	**-**	**-**	**5.67×10^{-9}**	**5.47×10^{-7}**	**3.51×10^{-6}**
Volume on western blot (μL)	4.50	3.60	4.95	4.50	3.60	1.13
Grey value	1.06	1.38	1.07	1.09	1.25	1.00
Detected activity* (normalized)	3.02	2.54	2.77	2.24	2.28	1.00
**Calculated activity# (normalized)**	**5.70**	**3.68**	**5.18**	**4.11**	**0.91**	**1.00**

Supplementary Table 1 | Purification and enzyme activity examination table. The enzyme activity of PbSRD5A during purification. The samples include: (1) whole cell crude extract; (2) re-suspended cell membrane after 1st round of ultracentrifugation; (3) solubilized solution in 1% DDM; (4) supernatant after 2nd round of ultracentrifugation, (5) protein eluted from Ni-column, and (6) purified protein after size-exclusion chromatography. HPLC was used to detect the 5a-DHP% converted from progesterone, using the same units of protein. Equally protein was quantified by calculating the grey level of western blot. The activities were calculated as: *Total activity* = *Total product* / *Reaction time*, *Specific activity* = *Total product* / (*Total protein* × *Reaction time*); *Calculated activity* = *Detected activity* / (*Sample volumes in an assay system* × *grey value* / *Volume loaded on western blot*), and normalized by sample #6.

	PbSRD5A
**Data collection**	
Space group	$C222_1$
Cell dimensions	
a, b, c (Å)	52.19 104.26 123.05
α, β, γ (°)	90 90 90
Resolution (Å)	19.36-2.00 (2.05-2.00) *
R_{sym} or R_{merge}	0.098 (0.84)
I / σI	12.80 (2.30)
Completeness (%)	99.60 (99.30)
Redundancy	6.60 (6.70)
**Refinement**	
Resolution (Å)	19.36-2.00
No. reflections	22971
R_{work} / R_{free}	19.50/23.27
No. atoms	
Protein	2008
Ligand/ion	200
Water	90
B-factors	
Protein	34.44
Ligand/ion	49.18
Water	41.2
R.m.s. deviations	
Bond lengths (Å)	0.01
Bond angles (°)	1.45

*Values in parentheses are for highest-resolution shell.

Supplementary Table 2 | Statistics of data collection and refinement for native PbSRD5A. One crystal was used for structure determination. Values in parentheses are for the highest resolution shell. Rmerge=ΣhΣi|Ih,i-Ih|/ΣhΣiIh,i, where Ih is the mean intensity of the i observations of symmetry related reflections of h. R=Σ|Fobs-Fcalc|/ΣFobs, where Fcalc is the calculated protein structure factor from the atomic model (Rfree was calculated with 8.71% of the reflections selected). a is reflections used for R and Rfree at low resolution bin. b is reflections used for R and Rfree at high resolution bin.

Category	HsSRD5A2	HsSRD5A1	PbSRD5A
NADPH binding residue mutations	N160D	N165	N159
	D164V	D169	D163
	R171S	R176	R170
	N193S	N198	N192
	E197D	E202	E196
	R227Q	R232	R226
	H231R	H236	H230
	Y235F	Y240	Y234
Catalytic site mutations	Q56R	Q59	Q53
	E57Q	E60	E54
	Y91D	Y95	Y87
Structural destabilizing mutations	A207D	A212	A206
	S210F	S215	T209
	P212R	Q217	A211
	Q126R	Q131	N124
	E200K	E205	E199
	P181L	P186	P180
	G183S	G188	G182
	S245Y	F250	E244
	R246Q/W	R251	R245
Helix breaking mutations	L20P	V23	S21
	L55Q/P	V58	A52
	H162P	H167	H161
	L224P	L229	L223
	H230P	E235	H230
Small to bulky residue (Destabilizing mutations)	G34R/W	G40	G33
	P59R	P62	P56
	G85D	A89	G81
	G115D	A120	A113
	G123R	G128	G121
	G158R	L163	A157
	G196S	G201	G195
	G203S	G208	G202

Supplementary Table 3 | Disease related loss-of-function mutations in HsSRD5A2. Disease related loss-of-function mutations in HsSRD5A2 and the corresponding residues in HsSRD5A1 and PbSRD5A are listed. The invariant residues and highly conserved residues between HsSRD5A2 and PbSRD5A are colored in red and purple, respectively.

Name	Forward primer (5'-3')	Reverse primer (5'-3')
PbSRD5A WT insert	GATGCTGGCAGCGGCCATATGGACCCGGATTTCCTGTATCGTT	ACCGCATGCCTCGACCTCGAGCTAAACCACAAACGGAATAACCGC
PbSRD5A N159A	TGGCTGGGCGCTGGCACTGCA	TGCAGTGCCAGCGCCCAGCCA
PbSRD5A D163V	CACAGCGTTGCGATCCTGCGT	AGGATCGCAACGCTGTGCAGGT
PbSRD5A R170S	TCGTCTGAGCGCGCCGGGTGAAA	TTTCACCCGGCGCGCTCAGACGA
PbSRD5A N192A	TTAGCTGCCCGGCATACCTG	CAGGTATGCCGGGCAGCTAA
PbSRD5A E196L	TGGGCCTGATCGTTGAGTGGT	ACCACTCAACGATCAGGCCCA
PbSRD5A R226Q	CTGTTTCCGCAAGCGCTGGCG	CGCCAGCGCTTGCGGAAACAG
HsSRD5A2 WT	GACGATAAGCTTATGCAGGTTC	TCATCTTTTAAGAATTCTGCAG
HsSRD5A2 Q56A	CCGCGCCGCCTGGTTCCTGGCAGAGCTGCCTTCCTTCGCGG	CCGCGAAGGAAGGCAGCTCTGCCAGGAACCAGGCGGCGCGG
HsSRD5A2 Q56L	CCGCGCCGCCTGGTTCCTGCTGGAGCTGCCTTCCTTCGCGG	CCGCGAAGGAAGGCAGCTCCAGCAGGAACCAGGCGGCGCGG
HsSRD5A2 Q56R	CCGCGCCGCCTGGTTCCTGCGAGAGCTGCCTTCCTTCGCGG	CCGCGAAGGAAGGCAGCTCTCGCAGGAACCAGGCGGCGCGG
HsSRD5A2 E57L	CGCCGCCTGGTTCCTGCAGTTACTGCCTTCCTTCGCGGTGC	GCACCGCGAAGGAAGGCAGTAACTGCAGGAACCAGGCGGCG
HsSRD5A2 E57Q	CGCCGCCTGGTTCCTGCAGCAGCTGCCTTCCTTCGCGGTGC	GCACCGCGAAGGAAGGCAGCTGCTGCAGGAACCAGGCGGCG
HsSRD5A2 Y91F	GGGCCTCTTCTGCGTACATTTCTTCCACAGGACATTTGTGT	ACACAAATGTCCTGTGGAAGAAATGTACGCAGAAGAGGCCC
HsSRD5A2 Y91D	GGGCCTCTTCTGCGTACATGACTTCCACAGGACATTTGTGT	ACACAAATGTCCTGTGGAAGTCATGTACGCAGAAGAGGCCC
HsSRD5A2 D164A	GGGAATAAACATTCATAGTGCCTATATATTGCGCCAGCTCA	TGAGCTGGCGCAATATATAGGCACTATGAATGTTTATTCCC
HsSRD5A2 R171A	CTATATATTGCGCCAGCTCGCAAAGCCTGGAGAAATCAGCT	AGCTGATTTCTCCAGGCTTTGCGAGCTGGCGCAATATATAG

HsSRD5A2 N193S	TACGTATGTTTCTGGAGCCAGTTTCCTCGGTGAGATCATTG	CAATGATCTCACCGAGGAAACTGGCTCCAGAAACATACGTA
HsSRD5A2 E197L	TGGAGCCAATTTCCTCGGTTTAATCATTGAATGGATCGGCT	AGCCGATCCATTCAATGATTAAACCGAGGAAATTGGCTCCA
HsSRD5A2 E197D	TGGAGCCAATTTCCTCGGTGACATCATTGAATGGATCGGCT	AGCCGATCCATTCAATGATGTCACCGAGGAAATTGGCTCCA
HsSRD5A2 R227Q	ACTTTGTTTCCTTGGGCTGGCTGCTTTTCACCACCATAGGT	ACCTATGGTGGTGAAAAGCTTGCAGCCCAAGGAAACAAAGT
HsSRD5A2 H231A	TGGGCTGCGAGCTTTTCACGCTCATAGGTTCTACCTCAAGA	TCTTGAGGTAGAACCTATGAGCGTGAAAAGCTCGCAGCCCA
HsSRD5A2 Y235A	TTTTCACCACCATAGGTTCGCACTCAAGATGTTTGAGGACT	AGTCCTCAAACATCTTGAGTGCGAACCTATGGTGGTGAAAA
HsSRD5A2 Y235F	TTTTCACCACCATAGGTTCTTCCTCAAGATGTTTGAGGACT	AGTCCTCAAACATCTTGAGGAAGAACCTATGGTGGTGAAAA

Supplementary Table 4 | List of primer sequences used in cloning and enzymatic assay.

Supplementary references:

1 Wallace, A. C., Laskowski, R. A. & Thornton, J. M. LIGPLOT: a program to generate schematic diagrams of protein-ligand interactions. *Protein engineering* **8**, 127-134, doi:10.1093/protein/8.2.127 (1995).

2 Krissinel, E. & Henrick, K. Inference of macromolecular assemblies from crystalline state. *Journal of molecular biology* **372**, 774-797, doi:10.1016/j.jmb.2007.05.022 (2007).

3 Ashkenazy, H. *et al.* ConSurf 2016: an improved methodology to estimate and visualize evolutionary conservation in macromolecules. *Nucleic acids research* **44**, W344-350, doi:10.1093/nar/gkw408 (2016).

4 Landau, M. *et al.* ConSurf 2005: the projection of evolutionary conservation scores of residues on protein structures. *Nucleic acids research* **33**, W299-302, doi:10.1093/nar/gki370 (2005).
