# Supplementary material for: Crystal structure of steroid reductase SRD5A reveals conserved steroid reduction mechanism
Source: Nat Commun. 2021 Jan 19;12:449. doi: 10.1038/s41467-020-20675-2 (PMC7815742; doi:10.1038/s41467-020-20675-2)
Supplement: Supplementary file 3 — Reporting Summary [file 41467_2020_20675_MOESM3_ESM.pdf]

nature research

Corresponding author(s): Ren, Ruobing
Last updated by author(s): Dec 7, 2020

Reporting Summary

Nature Research wishes to improve the reproducibility of the work that we publish. This form provides structure for consistency and transparency in reporting. For further information on Nature Research policies, see our Editorial Policies and the Editorial Policy Checklist.

Statistics

For all statistical analyses, confirm that the following items are present in the figure legend, table legend, main text, or Methods section.

n/a	Confirmed	
☐	☒	The exact sample size (*n*) for each experimental group/condition, given as a discrete number and unit of measurement
☐	☒	A statement on whether measurements were taken from distinct samples or whether the same sample was measured repeatedly
☒	☐	The statistical test(s) used AND whether they are one- or two-sided<br>*Only common tests should be described solely by name; describe more complex techniques in the Methods section.*
☒	☐	A description of all covariates tested
☒	☐	A description of any assumptions or corrections, such as tests of normality and adjustment for multiple comparisons
☐	☒	A full description of the statistical parameters including central tendency (e.g. means) or other basic estimates (e.g. regression coefficient) AND variation (e.g. standard deviation) or associated estimates of uncertainty (e.g. confidence intervals)
☒	☐	For null hypothesis testing, the test statistic (e.g. *F*, *t*, *r*) with confidence intervals, effect sizes, degrees of freedom and *P* value noted<br>*Give P values as exact values whenever suitable.*
☒	☐	For Bayesian analysis, information on the choice of priors and Markov chain Monte Carlo settings
☒	☐	For hierarchical and complex designs, identification of the appropriate level for tests and full reporting of outcomes
☒	☐	Estimates of effect sizes (e.g. Cohen's *d*, Pearson's *r*), indicating how they were calculated

Our web collection on statistics for biologists contains articles on many of the points above.

Software and code

Policy information about availability of computer code

Data collection	-SSRF (Shanghai Synchrotron Radiation Facility) beamlines BL18U1 with the structural data acquisition software integrated to the beamline.<br>-HPLC control software is Empower (version 3) and β-room collection software is Laura (version 6).<br>-Autodock Vina (version 1.1.2) is the program for molecular docking algorithm BFGS.<br>-Molecular weights in analytical ultracentrifugation analysis was calculated by SEDFIT (version 16.1c).
Data analysis	Structure:<br>Data index and integration: XDS http://xds.mpimf-heidelberg.mpg.de/<br>Data scale: AIMLESS https://www.ccp4.ac.uk/<br>Structure determination: PHENIX.PHASER http://www.phenix-online.org/<br>Model building: COOT https://www2.mrc-lmb.cam.ac.uk/personal/pemsley/coot/<br>Model Refine: PHENIX.Refine http://www.phenix-online.org/<br>Model Validation: PHENIX.Validation http://www.phenix-online.org/<br><br>Initial model:<br>MSA tool: hhblits https://github.com/soedinglab/hh-suite; jackhmmer http://hmmer.org/; blast https://ftp.ncbi.nlm.nih.gov/blast/executables/blast+/<br>Data integration:tgt_package https://github.com/realbigws/TGT_Package<br>Model building: trRosetta https://yanglab.nankai.edu.cn/trRosetta/<br>Model ranking: goap http://pwp.gatech.edu/cssb/goap/<br><br>Docking and MD simulation:<br>Protonation state prediction: http://biophysics.cs.vt.edu/<br>Docking inputs preparation: https://ccsb.scripps.edu/autodock/<br>Docking pose searching: http://vina.scripps.edu/

MD inputs preparation: http://www.charmm-gui.org/
MD simulation and analyses: http://manual.gromacs.org/documentation/2019/release-notes/index.html

HPLC data analysis: Prism 8.2.0

For manuscripts utilizing custom algorithms or software that are central to the research but not yet described in published literature, software must be made available to editors and reviewers. We strongly encourage code deposition in a community repository (e.g. GitHub). See the Nature Research guidelines for submitting code & software for further information.

Data

Policy information about availability of data

All manuscripts must include a data availability statement. This statement should provide the following information, where applicable:
- Accession codes, unique identifiers, or web links for publicly available datasets
- A list of figures that have associated raw data
- A description of any restrictions on data availability

Data supporting the findings of this manuscript are available from the correspongding author upon reasonable request. Structure factors and atomic coordinates of PbSRD5A protein have been deposited in the Protein Data Bank under accession code 7C83 [http://doi.org/10.2210/pdb7C83/pdb]. Initial models of PbSRD5A and two human SRD5As have been deposited in modelarchive with model IDs ma-xs1jw [https://www.modelarchive.org/doi/10.5452/ma-xs1jw] (PbSRD5A), ma-bfecj [https://www.modelarchive.org/doi/10.5452/ma-bfecj] (human SRD5A1) and ma-ib3wq [https://www.modelarchive.org/doi/10.5452/ma-ib3wq] (human SRD5A2). PDB entries (4QUV [https://www.wwpdb.org/pdb?id=pdb_00004quv], 4XU4 [https://www.wwpdb.org/pdb?id=pdb_00004xu4], 3COT [https://www.wwpdb.org/pdb?id=pdb_00003cot]) used in this study were downloaded from Protein Data Bank. Source data are provided with this paper.

Field-specific reporting

Please select the one below that is the best fit for your research. If you are not sure, read the appropriate sections before making your selection.

☒ Life sciences ☐ Behavioural & social sciences ☐ Ecological, evolutionary & environmental sciences

For a reference copy of the document with all sections, see nature.com/documents/nr-reporting-summary-flat.pdf

Life sciences study design

All studies must disclose on these points even when the disclosure is negative.

Sample size	No sample size calculation was performed. Enzymatic assays were performed in triplicate.
Data exclusions	Diffraction data sets that can not reach to 2.0 angstrom were excluded.
Replication	All diffraction data analyses have been reproduced at least three times. All attempts at replication were successful.<br>All HPLC analyses have been reproduced at least three times. All attempts at replication were successful.
Randomization	For structure refinement, 10% data were selected randomly for cross validation.
Blinding	Not applicable to macromolecular structure determination and analysis.

Reporting for specific materials, systems and methods

We require information from authors about some types of materials, experimental systems and methods used in many studies. Here, indicate whether each material, system or method listed is relevant to your study. If you are not sure if a list item applies to your research, read the appropriate section before selecting a response.

Materials & experimental systems

n/a	Involved in the study	
☒	☐	Antibodies
☐	☒	Eukaryotic cell lines
☒	☐	Palaeontology and archaeology
☒	☐	Animals and other organisms
☒	☐	Human research participants
☒	☐	Clinical data
☒	☐	Dual use research of concern

Methods

n/a	Involved in the study	
☒	☐	ChIP-seq
☒	☐	Flow cytometry
☒	☐	MRI-based neuroimaging

Eukaryotic cell lines

Policy information about cell lines

Cell line source(s)	Spodoptera frugiperda (Sf9) cell<br>HEK293T cell
Authentication	Sf9 cells were purchased from Sino-Bio (China);<br>HEK293T cells were purchased from the American Type Culture Collection (Manassas, VA)
Mycoplasma contamination	The cell lines were not tested for mycoplasma contamination.
Commonly misidentified lines<br>(See ICLAC register)	No commonly misidentified cell lines were used in the study.
